# The concept of tissue regeneration: Epistemological and historical enquiry from early ideas on the regeneration of bone to the microscopic observations of the regeneration of peripheral nerves

**DOI:** 10.3389/fcell.2022.742764

**Published:** 2022-10-26

**Authors:** Jean-Gaël Barbara

**Affiliations:** Sorbonne Université, UPMC Université Paris 06, Institut de Biologie Paris Seine (IBPS), Neuroscience Paris Seine, UMR CNRS 8246, Inserm 1130 & Sorbonne Paris Cité, Paris Diderot, Philosophie, Histoire, SPHERE, CNRS UMR7219, Paris, France

**Keywords:** nerve regeneration, nerve degeneration, waller, ranvier, ramón y cajal, schwann cell, myelin, peripheral nerve

## Abstract

This paper examines the epistemological history of physiological tissue regeneration theories from Antiquity to the present time focusing on early clinical observations, microscopic investigations of the 19th C. and molecular aspects of the regeneration of peripheral nerves. We aim to show underlying theoretical implications at stake over centuries, with an extreme diversity of local contexts, while slowly emerging ideas were progressively built in the framework of cell theory and that of molecular biology. The overall epistemological lesson is that this long history is far from finished and requires novel experiments and perspectives, as well as the careful inspection of its rich past, as a true scientific tradition, in order to better understand what is nervous regeneration and how we can use it in medicine.

## 1 Introduction

Regeneration is a biological concept with a long history to which new biotechnologies add a new medical dimension. However, this concept still lacks a general theoretical framework to bring nearer what is known about it in zoology (organ regeneration), medicine (tissue regeneration) and biology, together with its new molecular perspectives. Nevertheless, two present trends conciliate these perspectives. On the one hand, the molecular study of the regenerative capacities of animals (Franco et al., 2013) considers an evolutionary perspective, with the progressive loss of these capacities in higher animals (Bely and Nyberg, 2009). On the other, the study of the molecular mechanisms hindering or favoring these capacities aims at medically improving human tissue repair and regeneration. The convergence of these two types of molecular studies aims to achieve human tissue regeneration comparable to that encountered in non-mammal vertebrates.

Therefore, the molecular level of analysis of biological mechanisms of animal regeneration is of high interest for regenerative medicine (Carlson, 2007). However, the historical making of the concept of regeneration rather involved microscopic observations. At the present time, this is still essentially described phenomenologically as an ensemble of complex cellular mechanisms including cell dedifferentiation, cell proliferation, cell migration, redifferentiation and transdifferentiation, with cellular interactions among a large number of cell types and subtypes, including stem cells. As a matter of fact, a modern concept of regeneration should take all these cellular and molecular mechanisms into account, as well as their relations at multi-level scales.

The concept of tissue regeneration can be divided into “physiological regeneration” or the replacement of normal tissues such as nails, “hypertrophy” like the growth of liver tissue, “reparative regeneration” after the lesion of a tissue or an organ and the regeneration process of asexual reproduction. In this paper, we will present some historical and epistemological perspectives concerning the concept of reparative physiological regeneration in general and then focus on the model of peripheral nerve regeneration after section in vertebrates.

This regeneration implies cellular mechanisms leading to the development of a new functional tissue comparable to the initial tissue in the space, close to an inch, between the two cut ends of the nerve. This kind of regeneration is not considered epimorphic, since no blastem occurs, but it is said morphoallelic since it involves a massive tissue reorganization different from the formation of a scar. Such reorganization engages pre-existing cells which undergo profound modifications and dedifferentiation, before a stage of cell proliferation. Historically, in order to define such regeneration, the starting questions were 1) in which manners regeneration differs from scar formation, 2) whether a new tissue substance develops with a similar aspect and the same initial function as that of the injured tissue, 3) which anatomic elements are part of the regenerative process and which physiological mechanisms are involved.

It may come as a surprise to observe that some of these issues already arose during Antiquity, and that only very progressively clear answers were given (Table 1). The historical paths of the reparative regeneration concept show that both observations and reasonings followed winding roads, depending on the types of tissues (soft tissues or bone), and on the level of enquiry (tissue level, cell level, molecular level). For all these reasons, an epistemological and transhistorical reflection is necessary if we want to start bridging these issues together in a way which is not yet fully achieved at the present time, in particular concerning the connections between the cellular and molecular mechanisms of regeneration. For example, the study of some molecular signaling pathways involving specific receptors in particular cell types may still require the discovery of the cellular interactions at stake and of the cell subtypes responsible for the release of the signaling secreted molecule.

**TABLE 1:** Chronology of some ideas and concepts relative to the regeneration of tissues included in the article.

Date	Authors	Ideas & concepts
Antiquity	Hippocratic school (Greece) and Galen (physician, Roma)	No acceptance of the principle of bone regeneration in healing bone fractures. The faculty of regenerating new parts of the body is acceptable for flesh (not muscle) and fat
XVth C.	Ambroise Paré (surgeon, France)	Ideas on the formation of the “callus” of bones conceived of as a matter more solid and compact than the natural bone. Analogy with the drying sap from the cut end of a shoot of vine as the hardening of a mucilaginous mucus. The matters of the callus are considered as the “matters proper nourishing bones as well as flesh”
XVIIIth C.	François Quesnay (surgeon, France)	Acceptance of the idea of bone regeneration from a callus. The regenerative property of soft tissues is considered as a simple scarring process with the union of the cut parts
	Xavier Bichat (physician, France)	The callus of bones possesses dynamic and adaptive properties
	Paul-Joseph Barthez (physician) and the Montpellier vitalistic medical school (France)	Regeneration of all tissues is accepted as a general vital property
	Karl Rudolphi (anatomist, Germany)	Nerve regeneration seems perfect in the particular case of the limb regeneration of the Salamander
	William Cumberland Cruikshank (Surgeon, Scotland)	Observation of a regenerated nervous substance in post-morten examination in the experimental unilateral section of the vagus nerve in dog
	Felice Fontana (physicist and physiologist, Italy)	Demonstration of the nervous nature of the regenerated nervous substance in post-morten examination in the experimental unilateral section of the vagus nerve in dog, by the observation of specific nervous characters of the fibres with the microscope
XIXth C.	Henri Kühnholtz (physician, Montpellier, France)	Development of the ideas of Barthez on the “regenerative power” of soft tissues considered as a vitalistic force
	Different surgeons in various European countries	Numerous cases of scarred nerves with a successful return to normal function
	Carl Otto Steinrück (anatomist, Germany)	Correlation of the regeneration of nerve fibres observed histologically with the slow return to function of the cut nerves in kittens and frogs
	Theodor Schwann (anatomist, Germany)	Observation of new regenerated nerve substance containing fibrils not quite similar to the original ones in sectioned sciatic nerves of the frog after 3 months of regeneration
	Nasse, Günther, Schön and C.O. Steinbrück (anatomists, Germany)	Recognition of the formation of new axis-cylinders on both sides of the sectioned nerves
	Augustus Waller (anatomist, England)	Waller develops an original theory of nerve regeneration from the central stump of the cut nerve on the model of the embryonic development. Waller referred to “embryonic fibres” of the regenerative process. Waller stresses the importance of the elimination of the old tissue in the living central stump as a necessary condition for regeneration
	Moriz Schiff (anatomist, Germany), Jean-Marie Philippeaux & Alfred Vulpian (neurologists, France)	Attack of the Wallerian model of regeneration. Belief in the persisting of old fibres in the distal stump and in their important role in the regeneration process and return to function of the nerve. Philippeaux and Vulpian referred to this supposed regenerative process as a *peripheral autogenous regeneration*
	Louis Ranvier (anatomist, France)	Dismissal of the model of Schiff, Philippeaux & Vulpian. Adoption of the Wallerian model. Morphological study of the alterations of the medial and the distal stumps of the cut nerve and of myelin alterations. Description of how the nerve fibers fully disappear in the medial and distal stumps. Description of the disorganisation of Ranvier nodes et their reappearance after regeneration
XXth C.	Ramón y Cajal (anatomist, Spain)	Complete study of nerve degeneration and regeneration published in 1914
	John Newport Langley (England), Fernando de Castro (Spain), Giuseppe Levi (Italy), Jorge Francisco Tello Muñoz (Spain), Rita Levi-Montalcini (Italy)	Studies of the cellular mechanisms of regeneration in ganglia and cell cultures

## 2 Tissue regeneration observed with the naked eye

As soon as it became possible to describe the cellular mechanisms of tissue regeneration, around 1850, with modern microscopes, in the theoretical framework of cell theory, did a clear and rapid evolution occur in the history of the tissue regeneration concept. Quite rapidly, new models based on precise observations of cell interactions were built with mechanical and chemical explanations, as in bone formation or nerve tissue repair. In the preceding centuries, surgeons and also physicians closely inspected with the naked eye, or with rudimentary microscopes, the new tissues appearing after a lesion. But, as we shall see and explain, their conclusion was often that in both cases the new tissue did not derive from a real regeneration process, but from the formation of a scar.

### 2.1 The concept of regeneration in ancient medicine

Regeneration was first a philosophical concept which theology perpetuated as the moral and physical rebirth of an individual. In the medicine of Ancient Greece, the concept of flesh regeneration was closely associated with theories on generation (reproduction). In the Hippocratic school, as well as in the case of Aristotle until Galen, the regeneration concept did not evolve much. It was conceived of as depending on the faculties of the sperm of men formed and contained within the veins of the testis, but also potentially in all the veins of the body. For this reason, any vein was supposed to show a regenerative faculty which was justified by the fact that new veins could appear under some conditions, as in the case of varicose veins. Conversely, such view explained why all other tissues, including arteries, could apparently not regenerate. However, physicians and surgeons were nevertheless very well aware of the processes of tissue repair and remedies to be employed for scar formation.

### 2.2 French surgeon, Ambroise Paré, progresses in surgery and the idea of the regenerative process of bone formation after lesion

**FIGURE 1:**
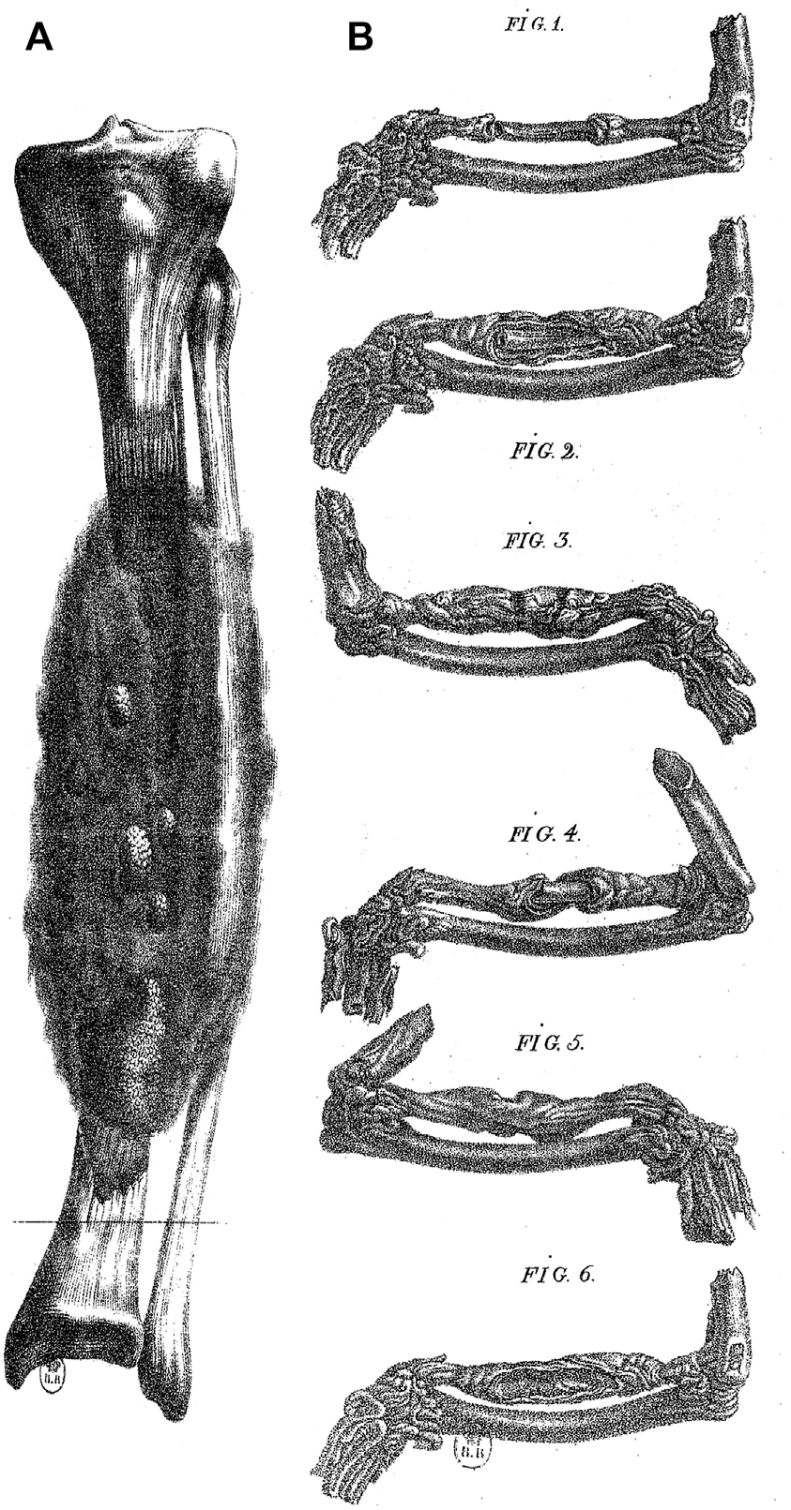
Bone regeneration in man and in the pigeon. **(A)** A case of bone regeneration in man (tibia). Adapted from plates 1 and 2, Charmeil, 1821. **(B)** Experiment by Charmeil of the destruction of the periosteum and endosteum in a portion of a bone from the pigeon wing. The destruction of the periosteum and endosteum induced necrosis followed by regeneration (Fig. 1). (Fig. 2 to Fig. 6), different stages of regeneration. Adapted from Charmeil, 1821.

In his treatise entitled *Recherches sur les métastases* (1821), French surgeon, Pierre Marie Joseph Charmeil (1782–1830), included the results of his “New experiments on the regeneration of bone”, where he studied regeneration from an experimental anatomopathological perspective in the pigeon, an animal model previously used in the 18th century (Charmeil, 1821; Figure 1). Charmeil was opposed to many past conceptions on regeneration, including those from the turn of the 19th century. However, Charmeil held in high esteem the surgeon Ambroise Paré (1510–1590), for his original views on the regeneration of tissues. His particularly detailed reading of Paré enabled him to detect some insights of Paré concerning regeneration. These include numerous pieces of advice of Paré concerning soft remedies to be employed to favor the formation of the new flesh invading the broken bone and slowly developing into a hard and white substance, without mentioning—at this stage—that this may be real bony substance (Malgaigne, 1840, book 16th, chapter 34). For the classical medical tradition, this new substance is that of a *callus*, a hardening tissue seen in the scar, for example closing the hole after trepanation, as already described by Hippocrates. Paré seems ready to admit with other surgeons and physicians that the callus is a scar, although the new substance appears as “more solid and compact than natural bone” (Malgaigne, 1840, book eighth, chapter 22). If we extend Charmeil’s reading of Paré to Paré’s Book 8 (chapter 41), we see the French surgeon comparing the formation of the callus with the drying sap from the cut end of a shoot of vine as the hardening of a “mucilaginous mucus” (*humeur spéciale*, *glaireuse*, *mucilagineuse*, Malgaigne, 1840). And when Paré gives his remedies for the formation of the callus, he describes it as a “hard substance […] made of what abounds from what nourishes the broken bone, which holds and agglutinates the bone together, and with time hardens so much that it becomes more solid and harder than the remaining non broken part of the bone” (Malgaigne, 1840, book 13th, chapter 3)^1^.

We thus see Paré more and more conscious that the formation of the callus is a process depending on the quality of the humours involved, the youth and the health of the patient, so that his concept of bone repair seems to get closer—in Paré’s mind—to a concept of a vital, regenerative and active mechanism which is in accord with our modern view. Indeed, Paré now uses the expression “the generation of the callus”. In this quite imperceptible shift in Paré’s writings, he comes to describe the vitalistic mechanism of the appearance of the callus from a “flesh which Nature produced upon, which being newly generated has the softness of the freshly clotted cheese […] [and] with time hardens, and forms similarly to the small grains of pomegranate, in which the serous pus is reddish, shiny, even, glutinous, not fetid, and then white” (Malgaigne, 1840, book 16th, chapter 34)^2^. In Book 13th (chapter 29), Paré gives further details on his conception of the “matters of the callus” as the “matters proper nourishing bones as well as flesh”. Thus Paré seems to get near to a regeneration concept, when he considers the callus formed from the substance of the “bone medulla and form the proper substance of bone” which makes the callus by “muddy and dirty sudation” (Malgaigne, 1840, book 13th, chapter 29)^3^. Therefore we have in Paré many of the elements of a first elaborate conception of bone regeneration with the formation of a new bony substance, made from bone matters, harder and as white as bone, which differs greatly from the past classical conceptions on scar formation.

### 2.3 Theories of bone regeneration in the 19th century

After Paré, discourses on bone regeneration will necessarily deal with the question of the nature of the newly formed substance and its origin, in accord with the doubts of Paré on the nature of the callus. However, the idea of bone regeneration progresses throughout the 19th century. We find it already defended at the very beginning of this century by leading French physician, Xavier Bichat (1771–1802). Charmeil is indebted to Bichat for his conception of the bone callus with dynamic and adaptive properties: “[The callus is] all the greater when the two bone ends are farther away, because the fleshy buds must travel through a greater space to meet and are therefore more expanded, and consequently have absorbed greater nutritive substance” (Bichat, 1801, *p*. 83). According to Bichat and his tissue classification, the formation of the callus would come from “compact and cellulous tissues, and from all parts of the divided surface in general” (Charmeil, 1821, *p*. 368)^4^. But Bichat’s conception is modern for his time, compared to the views of physicians in the following decades. Charmeil is indeed very critical of the following theories of bone regeneration where the callus is considered only as a product of the “medullary membrane” of the bone (endosteum) or from the periosteum, against the wider view of Bichat where soft tissues also are involved (Charmeil, 1821, *p*. 361).

In his own experimental work on bone regeneration, Charmeil demonstrated that the callus can develop in the broken pigeon’s wing although the medullary membrane and the periosteum were surgically removed (Figure 1). Charmeil concluded that all kinds of tissues are involved in bone regeneration with the “formation of buds on all divided surfaces, which is nothing but the expansion of the nutritive parenchyma connecting with the gelatine to transform successively into the cartilaginous state and then bone, a kind of development resulting from the vascular system, formative principle of any organic creation” (Charmeil, 1821, *p*. 369, *p*. 369)^5^. Charmeil’s theory illustrates the posterity of Bichat’s conceptions throughout the 19th century, on regeneration in particular, in which Charmeil adopts Bichat’s general conceptions of tissues and progressively foresees possible mechanisms implying the involvement and interactions of different types of tissues, getting closer to a modern conception of tissue regeneration.

### 2.4 Regeneration theories of soft parts before the 20th century

The issue of bone regeneration generated numerous polemics throughout the 19th century. However, a general model progressively emerged and was then rebuilt upon the new polemics concerning the cellular events at stake. The question of the regeneration of soft parts took more tortuous paths and led to a somehow inverted story compared to that of bone regeneration.

Indeed, during Antiquity, the Hippocratic school and Galen did not accept the principle of bone regeneration in healing bone fractures (Hippocrates, Aphorisms, Section 6, aphorism 19th), besides their knowledge on scarring processes. But the faculty of regenerating new parts of the body was acceptable for flesh (not muscle) and fat, as with warts and lipomas. Such a view on the regeneration of soft parts of the body survived until the 18th century and regeneration was accepted as vital property in the Montpellier (France) vitalistic medical school of Paul-Joseph Barthez (1734–1806).

During the 19th century, dissenting voices emerged, among them, those advocating for the ideas of French physician, François Quesnay (1,694–1774). Quesnay considered bone regeneration possible from a callus, but he considered that the supposed regenerative property of soft tissues only was a scarring process and a simple union of the cut parts (Quesnay, 1764, chapter 17, *De la régénération des chairs*, *p*. 255). Thus Quesnay adopted a position contrary to that of Antiquity.

Quesnay was also in opposition to Ambroise Paré and the Montpellier medical school of his time. He changed Paré’s analogy of the vine shoot with that of cut grasses the ends of which dry with no sap. This was justified by the fact that soft tissues, as nails, hair and warts do not regenerate from their cut ends, but from deeper parts. Quesnay thought that the dried tissues “extremely thin and weak” only formed a scar. He concluded that the idea of a real reproduction (regeneration) of flesh (soft tissues) was therefore untenable. Consequently, he excluded the idea of the regeneration of “sensitive vessels”, tendons and nerves. In his general perspective, any new substance formed after the lesion of a bone, skin, fat, membranous parts or brain differed from the original one, on the basis of some rudimentary microscopic observations (Quesnay, 1764, *p*. 261)^6^. From an epistemological standpoint, no precise norm of substance semi-similarity could be defined precisely in the regeneration theories and such a semi-sameness of the new tissues could as well justify that they simply formed a scar rather than a regenerated tissue. It was more or less a question of standpoint before precise microscopic investigations.

Thus, the question whether a scar included a newly generated substance and implied regeneration, as with skin or veins, with a return to normal physiological functions, remained for long^7^. This was the state of the regeneration issue, when Henri Kühnholtz, (1794–1877), a French physician from the Montpellier medical school, published his *Mémoire* in the *Bulletin de l'Académie Royale de Médecine* in 1856, where he defended the ideas of Barthez and his concept of the “regenerative power” (*pouvoir régénérateur*) of soft tissues, considered as a vitalistic force (Kühnholtz, 1841).

Kühnholtz based his theory on the widely accepted bone regeneration concept extended to soft tissues. He also defended the idea of Charmeil according to which all kinds of tissues participate in bone regeneration, with the consequence that soft tissues involved in the process shared the vital regenerative faculty.

The metaphors of tissue regeneration evolved similarly. While Quesnay refused that of the mason filling gaps of new constructions with mortar, Kühnholtz used the metaphor of the tailor, since the tailor does not only sew torn pieces of clothes, but he can also bring new pieces of tissue, not quite similar, but close enough, and fulfilling a similar function. This is how Kühnholtz saw the regeneration of soft tissues, where a new tissue replaces the original one, with the same general aspect, but not entirely identical, and explaining a return to normal physiological function, along with the perspective which developed throughout the 19th century in various contexts.

## 3 Theories of peripheral nerve regeneration

The theory of the regeneration of soft tissues of Kühnholtz was based on a synthesis of clinical and experimental observations made on various kinds of tissues, including the nervous tissue. In the midst of 19th century, the long history of nerve surgery after lesion recorded numerous cases of scarred nerves with a successful return to normal function mainly from the end of the 18th century onwards (Holmes, 1951; Ochs, 1977)^8^. It was possible to think that such a return to function was due to the filling of the empty space between the two cut ends of the nerve by a new nervous substance. Progressively, as the techniques of nerve sutures improved, more and more physicians acknowledged nerve regeneration after several successful and spectacular cases.

This situation fostered surgeons and anatomists to perform experimental animal studies of nerve regeneration in the 18th century. To the extent that nerve regeneration seemed to appear perfect in the particular case of the limb regeneration of the salamander to Berlin anatomist, Karl Rudolphi (1771–1832), although he personally believed it impossible in warm blooded animals (Rudolphi, 1825, *p*. 87–88). However, many investigators, performing experimental studies on Vertebrates, including pigeons, kittens and puppies, as well as in humans, accepted a limited nervous regenerative property, after meticulous visual inspection of the newly formed nervous substance. Some scientists, as Felice Fontana (1730–1805), also used basic microscopes for this purpose.

### 3.1 The studies of nerve regeneration before Augustus Waller

**FIGURE 2:**
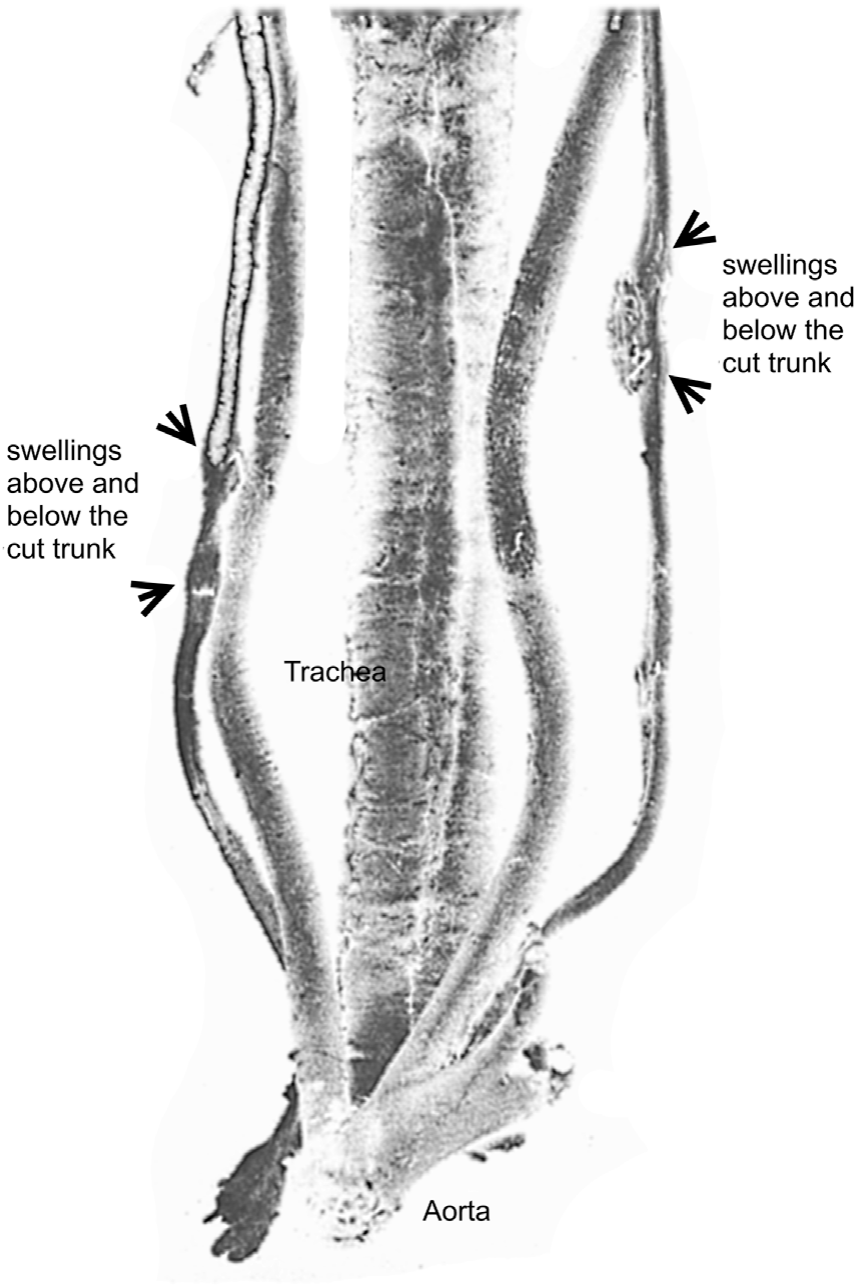
The preparation of a nerve suture followed by regeneration in a dog by Cruikshank at the Hunter’s museum. Adapted from Ochs, 1977. Illustrated from a posterior view, the preparation shows the aorta, the trachea, the right vagus nerve and the left vagus nerve both reunited after section by regeneration.

As early as 1776, Scottish anatomist, William Cumberland Cruikshank (1746–1800), performed the experimental unilateral section of the vagus nerve in a dog and he observed a regenerated nervous substance in post-morten examination (Figure 2). His study was not accepted for publication by The Royal Society (London) until 1795, when it was finally edited together with new and similar results by British surgeon, John Haighton (1755–1823) (Ochs, 1977, *p*. 261; Holmes, 1951, *p*. 46–49; Cruikshank, 1795; Haighton, 1795). It was clear for Cruikshank that the regenerated substance was nervous in nature, but not to Felice Fontana who observed the anatomical piece during his visit to Hunter in London. However, Fontana reproduced the experiment and performed microscopic observations on fresh regenerated tissues, and demonstrated their nervous nature by the observation of specific nervous characters of the fibres which appeared with characteristic bands with his microscope (Clarke and Bearn, 1972; Ochs, 1977, *p*. 264)^9^, although he previously felt that the union of the cut ends of a nerve was rather a scarring process^10^.

However, the regeneration process during these initial animal experiments was incomplete and with no return to the normal function of the nerve. Return to function was inferred by the survival of the animal; if the animal did not die until long after section, the sectioned vagus nerve was believed to function again normally, as it was thought that this nerve was essential to life. From an epistemological point of view, these issues imply two distinct perspectives. On the one hand, microscopic studies, before those of Waller, were performed to demonstrate the nervous nature of the new tissue between the two cut ends of the nerve without any functional norm of regeneration (Cruikshank, Fontana, Haighton). On the other hand, clinical evidence in humans demonstrated the precise timeline of physiological return to function using nerve sutures, but without the possibility to firmly establish the nervous nature of the regenerated tissue, except in rare cases after autopsy (Kühnholtz, 1841, *p*. 29–33; Holmes, 1951, *p*. 52–53, 56).

Not until 1840, was it possible to establish the strict correlation of the microscopic events of the regeneration of nerve fibres observed histologically with the slow return to function of the nerves cut in kittens and frogs, by Carl Otto Steinrück (1817-?) (Ochs, 1977, *p*. 266–267; Steinrück, 1838), sometimes after more than a year in order to get full regeneration.

Between the observations of Fontana and those of Waller, much progress was made in the histological techniques, notably using new dyes, and detailed microscopic observations were possible. The classical chronology of such studies includes those of Swiss physician, Jean-Louis Prévost (1790–1850), in 1826, showing new nerve fibres elongating from the central part of the cut nerve towards the medial part of the section (Prévost, 1826; 1827; Müller, 1835), or those of French physiologist, Pierre Flourens (1794–1867) (Flourens, 1828; 1835). Theodor Schwann (1810–1882) also made similar observations while an assistant to Johannes Müller, on sectioned sciatic nerves of the frog after 3 months of regeneration, showing the new regenerated substance contained fibrils, not quite similar to the original ones (Müller, 1838, *p*. 421). In fact, for Kühnholtz, this semi-similitude was an element of his theory of tissue regeneration which we now refer to as “reparative regeneration” different from the simple growth of hair and nails. Müller accepted the value of Schwann’s observations and considered them new, because he felt previous investigators, such as Fontana, Prévost, Michaelis, Meyer or Tiedemann, could not have observed new fibres since the animals were sacrificed for observations well before regeneration was believed to have occurred^11^. Consequently, Müller suggested his assistant Schwann had first demonstrated in 1830 the reproduction (regeneration) of a new nerve substance formed by fibrils crossing the medial part of the cut nerve. Müller was probably wrong in granting priority to his school^12^.

### 3.2 The importance of studying nerve degeneration before the regenerative process from Arnemann to Augustus Waller

The studies on nerve regeneration of Augustus Waller (1856–1922) opened a new era with his microscopic skills in part acquired with French microscopist, Alfred Donné (1801–1878) and later with German physiologist, Julius Budge (1811–1884). Waller made systematic cytological observations of degenerating and regenerating nerve fibres in the transparent tongue of the living frog, in the framework of cell theory and later of the neurone theory.

Among other histologists from the second half of the 19th century working on these issues, French anatomist, Louis Ranvier (1835–1922), rightly noticed, as we will see from several examples, that any new theory of nerve regeneration necessarily relied on the initial interpretations of degenerating nerve fibres observed in a cut nerve^13^.

For this reason, in the study of nerve regeneration, it became central to study the intimate mechanisms taking part in the medial stump of the cut nerve and the peripheral end, when it was widely acknowledged that the degeneration of nerve fibres and their loss were phenomena enabling regeneration.

In the letter he sent to the French *Académie des sciences* in Paris on November 23rd of 1851, Waller wrote that the issue of nerve regeneration had virtually made no progress since the work of Felice Fontana until his own (Waller, 1852a, *p*. 3). While proving Ranvier’s judgment was right, he ascribed that situation to the fact that the degeneration of the distal stump of the cut nerve had never been correctly observed, when he personally saw this mechanism as the key to the understanding of the process of regeneration (Waller, 1852a, *p*. 4)^14^.

However, Waller was not fair^15^. When submitting his paper to the *Royal Society* (Waller, 1850), William Sharpey (1802–1880) reviewed the paper and required Waller to quote German authors, Hermann Nasse (1807–1892), Augustus Fridericus Günther (1806–1871) and Matthias Johann Albrecht Schön (1800–1870), who had previously described the degenerative distal nerve stump (Sykes, 2004, *p*. 35). If Waller finally did so, he did not clearly mention their observations compared to his own and for a while they fell into oblivion.

In order to understand the new theories of nerve regeneration in the 19th century, it is necessary to define the various contexts of study of the degenerating processes before Waller. One of the first enquiries was made by surgeon and professor of medicine in Göttingen, Justus Arnemann (1763–1806), well-known for his nerve sutures. Arnemann fought the ideas of Cruikshank and Haighton and he did not accept the concept of a regenerative nerve substance (Holmes, 1951, *p*. 46, 50, 52; Arnemann, 1786; Arnemann, 1787)^16^. However, he described the degenerating distal end of a cut nerve with the idea of proving that regeneration was not possible, including the regeneration from that end of the section.

The famous histological studies of Nasse (1839), Günther and Schön (1840), before those of Waller, proving the degeneration of the distal stump, were primarily aimed at the understanding of the kinetics and the anatomical determinism of the loss of, and return to, function of the cut nerve in an anatomo-pathological perspective (see for example Jaccoud, 1864, *p*. 166). The works of authors after Waller quoted these studies when they realized their interest which was eclipsed by the success of Waller’s studies.

Following Waller’s studies on degeneration, Ranvier added many microscopic details which he mentioned in his *Leçons sur l’histologie du système nerveux* (Hernandez Fustes et al., 2019), including the “progressive alterations of the nerve tubes” of the distal stump of a nerve cut in a frog or a rabbit, with “myelin segmentation”, “fatty granules” merging into numerous droplets more abundant in the medial part of the section, but which were partly in agreement with Nasse (1839, *p*. 409–413). For Ranvier and his contemporary investigators, the “Schwann substance” (myelin) of nerve fibres disintegrated into ever smaller fragments which aggregated into ovoid droplets, a description completing Waller’s initial ones.

Concerning this process, Waller considered likely the possibility that eventually all nerve fibres degenerated on both sides of the section. Thus it became generally acknowledged that Nasse, Günther, Schön and C.O. Steinbrück (1838) recognised the formation of new axis-cylinders on both sides of the section without distinction (see for example Ziegler, 1895, *p*. 257). At this stage, many other cellular theories of nerve regeneration occurred in various contexts of study and various theoretical frameworks, sometimes following cellular models of bone regeneration with the involvement of different tissues, including the “cellular tissue” and blood cells.

### 3.3 The theory of regeneration of peripheral nerves of Augustus Waller

In his studies, Waller progressively modified the cellular theory of nerve regeneration of Nasse, Günther, Schön and Steinbrück, on an essential point: newly formed fibres only came from the central stump considered alive because of the trophic action of the nerve centres. Such trophic influence was later interpreted as the functional connection of nerve fibres with the body of nerve cells, when, in his experiments on the dorsal and anterior roots of the spinal cord, Waller showed that the trophic action was in fact due to the “ganglionic cells” of dorsal root ganglia or to the “motor nerve cells” of the anterior horns of the spinal cord.

**FIGURE 3:**
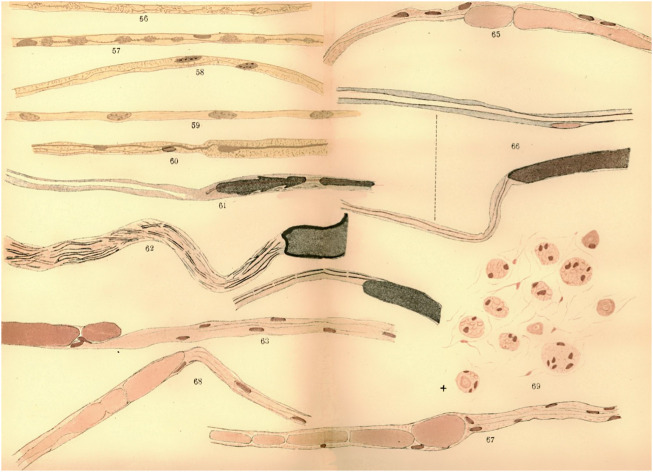
Microscopic observations from a regenerated nerve of a dog. The central end of the median nerve was sutured to the peripheral end of the ulnar nerve, and long pieces were then removed from the peripheral stump of the median and the central stump of the ulnar, to prevent the possibility of union. Newly formed fibers were observed (57, 58), as well as an embryonic fiber (59). Adapted from Howell & Huber, 1892.

The new fibres from the central stump were interpreted by Waller as the generation of novel embryonic nerve fibres. Ranvier interpreted Waller’s conception by the fact that Waller agreed with Nasse, Günther, Schön, and Steinbrück, about the total disappearance of degenerating nerve fibres near the section. New fibres were then necessarily seen as novel entities. But the idea of the total disappearance of the fibres in the central stump is wrong, since altered nerve fibres do stay alive in this part. But, for these authors, the new fibres originated elsewhere from the edges of the nerve centre, as in embryonic development (Figure 3).

Actually, Waller’s conceptions on nerve regeneration were developed prior to his idea that the trophic action on nerve fibres was due to ganglionic cells. In his early studies on the alterations of cut nerve ends in the tongue of the frog^17^, Waller already made observations indicating 1) a protective role of the nerve centres in the nervous disorganisation, 2) alterations in the cut nerve fibres, but also 3) an arrest of these phenomena after the union of the two cut nerve stumps. The first point can be found in the paper of 1850 with the observation that the alterations of the fibres are progressively less frequent following the nerve tract towards the brain^18^. Curiously, the third point, present in the oral presentation in its abstracted version (Waller, 1851, *p*. 925), does not appear anymore in the full published paper. Perhaps the hypothesis of the oral presentation was rejected by the reviewer. It concerned a quite unequivocal interruption and return of a possible role of the centre in the nutrition of nerve fibres by means of the nerve impulse, which Waller justified by the fact than when the cut nerve ends are united, the nervous disorganisation stops and the nerve fibres return to their normal state.

Thus, in 1849, when Waller writes his communication, he already admits a trophic role of the nerve centres. In 1850, this view and his observations enabled him to write, as we mentioned above, in his letter to the *Académie des sciences* in Paris, that the issue of degeneration had not progressed since Fontana, justifying this statement by the discovery that since nerve fibres degenerate completely, new ones necessarily appeared *de novo* ^19^.

There are several reasons why Waller referred to embryonic development in explaining regeneration. In his microscopic studies, Waller paid attention to the state of the structures he was studying in human embryos, as in the case of the papillae of the tongue (Waller, 1849a; Waller, 1849b). He had a good knowledge of embryonic tissues and he was able to compare the thin and pale new fibres to those observed in embryos. Waller observed precisely the greyish aspect of the new fibres, their intimate contact, the lack of double contours (myelin) (Waller, 1852b, *p*. 393–394)^20^. For Waller, regeneration was the start of a new phase of development after the complete removal of old fibres^21^. In his description, Waller goes as far as to use the expression of the “embryonic fibres” of the regenerative process, noticing that their observation preferentially requires the use of young animals in which regeneration is faster, as Waller checked with electrical stimulations *in vivo* (Waller, 1852b, *p*. 394).

Waller describes regeneration as an embryonic process with the appearance of nuclei (Schwann nuclei, i.e., Schwann cells) and the double contours of nerve fibres (myelin) deriving, in his opinion, from the neurilemma. In a later work focussing on the medial part of the cut nerve, Waller considered the central stump as normal, and the distal stump as highly disorganised. He noticed how the medial part of the nerve progressively becomes a proper medium for regeneration. He noted that while old nerve fibres disappear, capillaries invade the medial part which becomes less opaque (because the debris of the nerve fibres are removed by white blood cells), whereas the distal stump is very dark, lacking capillaries, with numerous non resorbed granulations (Waller, 1852c, *p*. 676). Waller concludes with the important fact that the speed of absorption and elimination of the granulations of the old tissue in the living central stump is a necessary condition for embryonic development when new fibres chase old used products (Waller, 1852c, *p*. 676).

We can now give a further interpretation of Waller’s use of an embryonic developmental model of regeneration. It is possible to link the trophic role of which Waller credits the nerve centres, and then the ganglionic cell, during regeneration, to the role played by the nerve cell during embryonic development, according to the law of unilateral growth, as expressed—for example—by Albert von Kölliker (1817–1905) (Kölliker, 1852)^22^. Therefore, this conception of embryonic development accords with the concept of regeneration of Waller and his law of degeneration. It is such a concordance which enabled him to write: “Therefore, it is demonstrated that when a nerve is cut […], its new fibres […] develop from the centre to the periphery and not from the periphery to the centre. I think it would be superfluous to examine the issue whether these fibres of the adult or those of the young animal develop in the same manner as in the embryo, since it is impossible to admit that Nature operates differentially in both cases” (1852c, *p*. 676–677)^23^.

We conclude that the theory of regeneration of Waller has been an early and important source of reflections already in his early studies on nervous degeneration and the trophic role of nerve centres and nerve cell. On this basis, Waller developed an original theory of nerve regeneration from the central stump of the cut nerve on the model of the embryonic development taking place in a milieu where old debris of the degeneration were cleared out, in accord with the previous conception of a nerve substance originating from a former one, and in opposition to the idea that the union of the cut ends of a nerve is a simple scarring which explains the return of function in humans^24^.

### 3.4 The new theory of nerve regeneration of Schiff, Philippeaux-Vulpian and Remak

After Waller issued his first publications, other scientists followed in his path, also following his advice to search for the mechanisms of regeneration by carefully examining the degeneration process of the distal end of the cut nerve. Among those, one of the earliest was German physician, Moriz Schiff (1823–1896) in 1854, followed by French physiologist, Jean-Marie Philippeaux (1809–1892), and his pupil Alfred Vulpian (1826–1887), in 1858^25^.

Schiff, then director of an ornithology department in an institute of natural history^26^, was interested in the hypoglossal nerve of the tongue of the frog (Schiff, 1853), an issue much debated in the physiological lessons of Vulpian. This study already included a case of nerve suture, but in the following year (1854), Schiff pursued this line of research when he reproduced the experiments and observations of Waller on nerve degeneration. He was able to present his findings at the *Académie des sciences* in Paris thanks to French ornithologist, and nephew of French Emperor Napoleon Bonaparte, Charles-Lucien Bonaparte (1803–1857) (Schiff, 1854). The general tone of the communication by Schiff is quite aggressive and shows great confidence, when he ridicules Waller’s fascination for his concept of embryonic fibres: “[…] in some cases where M. Waller revealed the return of the functions [of the cut nerve] by galvanism, he indeed saw true regenerated fibres, but he did not pay attention to them, too much preoccupied as he was by his putative embryonic fibres” (Schiff, 1854, *p*. 451, *p*. 451)^27^.

Schiff allowed himself this criticism of Waller’s observations and theory because in his mind clinical observations of a return to function of an injured nerve in humans sometimes occurred before the supposed regeneration of new fibres invading the distal stump from the central stump (Schiff, 1854, *p*. 449)^28^. In fact, anatomist and historian of science, specialist of nerve sutures, William Holmes, gave at least three reasons to understand the facts reported by Schiff with no opposition to the regeneration theory of Waller in its general lines (Holmes, 1951, *p*. 59): 1) the growth of new fibres may be very early and fast in fully mastered animal experiments (specially in young animals); 2) the delay of appearance of the fibres is overestimated by the difficult observation of thin new fibres without dyes; 3) the return of function of the injured nerve may also be due to a reinnervation from adjacent innervated structures and not to regeneration.

From his apparently strong standpoint, Schiff was led to reinterpret the observations of Waller in a totally new direction. Schiff concentrated his observations on the distal end of the nerve where he noticed persisting membranes (Schwann sheaths) around granulations, which he interpreted as the persistence of old fibres with their primitive axis-cylinder. Schiff criticized Waller’s interpretation of thin and pale new embryonic fibres, and decided that the state of the old fibres he saw represented an ultimate degenerated state of old and still lasting fibres.

Philippeaux and Vulpian reproduced these observations, with the same error, since the axis-cylinders were in fact absent. Furthermore, they extended Schiff’s interpretation and theory. They imagined that the apparently persisting old fibres of the distal stump were central in the regeneration process and return to function of the nerve, when these fibres presented a double contour (myelin) again. Philippeaux and Vulpian referred to this supposed regenerative process as peripheral autogenous regeneration (Philippeaux & Vulpian, 1859a; 1859b; Ochs, 1977, *p*. 271), since it did not require any trophic action of the central stump. The success of this theory was such that Louis Ranvier noted in his lessons that Waller himself changed his mind and agreed with Vulpian whom he knew personally (Ranvier, 1878b, *p*. 74).

Ranvier points out the fact that this theory gained further credit when Robert Remak (1815–1865) supported it in order to explain an incidental observation in a regenerated nerve of a rabbit from an anatomical preparation made for him by one of his former pupil, the son of German physician Friedrich Jacob Behrend (1803–1889). Remak made the then peculiar observation of new fibres inserted into old tubes (Schwan cell tubes lacking the degenerated fibre) containing characteristic granules (Remak, 1862). It is interesting to note that Waller had never observed this, since he writes: “In all my work on that issue, I never saw any new fibre inside an old tube” (Waller, 1852a, *p*. 4). Remak was led to interpret the origin of the new fibre he saw and, in his mind, it could not originate *ex nihilo* or from the central stump, as suggested by Waller. Therefore, Remak joined the advocates of the theory of Schiff, Philippeaux and Vulpian. Since then, with this observation of Remak, it had become almost impossible not to acknowledge that these new axis-cylinders could only derive from the residues of the old degraded axis-cylinders contained in the old tubes of the distal end of the nerve. On some occasions, Remak also observed several fibres in the same tube, and he imagined that the old fibres could undergo a hypertrophy and consequently a longitudinal division with the formation of two or more fibres.

Thus, the theory of regeneration of Waller gradually gave way to this peripheralist autogenic theory of nervous regeneration. But after all, at that time, the regenerative property of nervous tissue, supposedly common to all soft tissues, could not be excluded from the distal end of the lesioned nerve, especially in young animals, as Schiff commented as a possible explanation of Vulpian’s experiments, and in accord with spontaneous nerve ends union quickly after section, for example in the nerve grafting animal experiments of Flourens and later Paul Bert.

### 3.5 The theory of regeneration of Louis Ranvier

The observations of nerve degeneration and regeneration by Louis Ranvier (1835–1922) and his theoretical considerations are often forgotten or overlooked. But they are of prime importance as Spanish histologist, Santiago Ramón y Cajal, acknowledged (Barbara, 2007), especially in his book on degeneration and regeneration published in 1913 (Ramón y Cajal, 1914; De Felipe & Jones, 1991, part II). Cajal valued Ranvier’s work and ideas because they provided a very careful examination of the degeneration of lesioned nerves, chronological and semi-quantitative, and also because they paved the way to the modern concept of the “Schwann cell”, an intuition of Ranvier recognised by Cajal as a mark of genius^29^ (Barbara and Foley, 2022, forthcoming).

In his book, when Ramón y Cajal reviews the general and modern aspects of degeneration and regeneration of nerves, he mentions Waller, Ranvier, Vanlair, Nothafft, Stroebe, Ziegler, in that order, and often omitting Waller about the issues which he had not addressed. Sometimes he refers to the old “theory of Waller and Ranvier” attacked by the “polygenists”, Schiff, Philippeaux, Vulpian, Remak, because the theory of autogenic nerve fibres states that they can grow from different locations, either the nerve cell or the periphery. And Cajal finally points out the role of the memoirs of Belgian physician and anatomist, Constant François Vanlair (1839–1914) (Vanlair, 1882a; Vanlair, 1882b; Vanlair, 1885; Vanlair, 1893a; Vanlair, 1893b), establishing what Cajal refers to as the “modern theory of Ranvier and Vanlair”. Ranvier has indeed been an ardent defender of the theory of Waller at times when it was under strong attacks and almost fully demolished. Thus Ranvier belongs to the group of ancient histologists, but we can also credit him, as Cajal did, for establishing the first modern theory of degeneration and regeneration of nerves.

The systematic observations of Ranvier clearly refuted without any need for further discussion the interpretation by Schiff, Philippeaux-Vulpian and Remak. In his lessons on the nervous system, Ranvier addresses Remak this strong criticism: “[…] Had [Remak] made a single transversal section of the distal [peripheral] stump, from the fourth to the 10th day after section, he would have recognised that the peripheral axis-cylinders are not preserved” (Ranvier, 1878b, *p*. 45). Ranvier also mentions that he was able to convince Vulpian of his error; and Vulpian published the reasons why he was mistaken, while once again Ranvier could not agree with Vulpian, on the faulty interpretation of his error (Ranvier, 1878a, *p*. 274–275). The point is that Vulpian wrongly considered the staining of the inner part of the empty tubes of the Schwann sheath as an evidence of the persistence of some elements of the degenerated fibres.

It is impossible to present here all the novel aspects Ranvier brought on the study of degeneration and regeneration related to other publications of his time. Cajal’s review is of great help, among other studies by contemporaries, to evaluate the reception of Ranvier’s discovery and the inception of his novel views. Among other things, Ranvier studied in great detail the morphological alterations of the medial and the distal stumps and described myelin alterations, fragmentation and disorganisation into debris and fatty elongated (ovoid) droplets, among altered pale and granulous fibres. When describing the hypertrophy and multiplication of the Schwann nuclei, Ranvier considered that these phenomena were due to the arrest of an inhibitory trophic action of the nerve fibres which was their common cause^30^. These phenomena had previously been described, but Ranvier demonstrated that, prior to the multiplication, a single Schwan nucleus correlated with a single internode segment of the sheath of Schwann of a given fibre^31^. This functional organisation was lost during the multiplication phase and it recovered after regeneration with a distinct internode length taken as an indication of the regenerative process. Ranvier described how the nerve fibres fully disappear in the medial and distal stumps, leaving “cords” made up of granulous protoplasm (cytoplasm of the Schwann cells), while the debris of the altered myelin appear absorbed and cleared out by white blood cells [lymphocytes of Ranvier^32^] and blood circulation.

Concerning this last point, it is remarkable to note that Ranvier performed a physiological experiment demonstrating this possible mechanism, as a former physiological assistant of Claude Bernard, and an early advocate of experimental histology, based on Magendie^33^, Bernard and also German histologists (Duchesneau, 2019)^34^, at the frontiers between physiology and histology (Barbara, 2012, *p*. 91–108, 2016, 2017). In the line of similar experiments by German physician Friedrich Daniel von Recklinghausen (1833–1910), Ranvier prepared a solution of myelin extracted from the spinal cord of a Guinea pig which he injected into the peritoneal cavity of another animal. When he collected a sample of the liquid from that cavity with a serynge, after few hours, he showed that the “lymphatic cells” contained typical myelin droplets proving their faculty to absorb and clear out myelin in the external milieu (Ranvier, 1878a, *p*. 300).

In the distal stump only, Ranvier further observed that after multiplication the Schwann nuclei decrease in number while the sheaths of Schwann become thinner and flatten, forming a cavity where new fibres will grow from sproutings coming from the central stump. In this stump, degeneration stops quite rapidly and the nervous fibres are not much altered, becoming thinner and undergoing also a kind of hypertrophy with sometimes the formation of large globular masses, that Cajal later interpreted as large clubs due to the degeneration of lost fibres which did not make their way to the distal stump.

Ramón y Cajal was very aware that almost all observations by Ranvier were relevant to myelinated fibres stained with osmic acid, and in rare cases with carmine. So, most of Ranvier’s studies on the behaviour of new nerve fibres were made after myelinisation. Therefore, as Cajal notes, Ranvier overestimated the delay of reinnervation of the distal stump (almost a month), and Cajal could later see them as early as on the 10th day after section. Nevertheless, Cajal never concealed his admiration for Ranvier who took the greatest advantage of the techniques which he used and further developed^35^. Ranvier observed a correlation between a Schwann cell and an internode segment and the loss and return of this correlation after degeneration and regeneration, respectively. Moreover, Ranvier argued that regenerating fibres are truly new since the length of their interannular segment differs from that of the old fibres. With these new concepts, Ranvier established cytological norms useful in the follow up of the processes of degeneration and regeneration, which norms were based on the morphological changes of Schwann cells (Barbara, 2007).

Using osmic acid, Ranvier also managed to describe peculiar spiral structures of nerve endings not reaching their target, some fibres of a bundle entering old tubes while others did not, and some fibres from two separate bundles crossing and passing from a bundle to another. But what struck Cajal most was Ranvier’s view of the Schwann nuclei and the Schwann sheath as a cellular unit, when Cajal writes: “Ranvier had an intuition of genius when he put forward the notion of the interannular segment as a vast cellular unit within which are contained the nucleus, myelin, and axon. The modern histologists have confirmed this doctrine in all its essentials” (Barbara and Foley, 2022; Barbara & Boullerne, 2020; De Felipe & Jones, 1991, part II, *p*. 44).

Finally, Cajal pays tribute to Ranvier for his strategy of “anatomical deduction”, for example when Ranvier hypothesized, as Vanlair and Cajal himself did later, that the new fibres possess an intrinsic property of growth and a property to find their path both randomly and following the “path of least resistance”. Although Cajal rejected this idea, and favored chemical and other mechanical explanations, he chose to attach Ranvier’s explanatory strategy to the chronology and history of alternative theories of chemotactism, neurotropism by Forsmann in 1898 and others, all of them summarized by Martin Heidenhain (1864–1949, Hedeinhain , 1911).

We conclude that Ranvier revolutionised the theory of degeneration and regeneration of nerve following Waller by establishing the foundations of the modern view which further developed at the turn of the 20th C. around the conception of the Schwann cell. One of the many reasons why Ranvier succeeded was that he studied degeneration simultaneously with Waller and had a background on bone and epithelia. He was thus prepared to study the functional implications of several cell types and their mutual interactions, as he was when looking for cells able to clear out myelin fragments among “lymphatic cells, “conjunctive cells”, or “endothelial cells” either normal or modified by inflammation. Ranvier was able to combine his meticulous techniques and precise observations, with the general cellular perspectives of Rudolph Virchow. Ranvier also developed these perspectives in his “general anatomy” which he applied with success to his anatomopathological and histophysiological cellular study of the degeneration and the regeneration of injured nerves.

## 4 The theory of regeneration of Ramón y Cajal and contemporary molecular perspectives

The doctrine of Ranvier is a midpoint between that of Waller and the great synthesis of Ramón y Cajal (1914). In the same way, we may say that the work of Cajal represents, itself, a midpoint between classical histological investigations and the new paths of the modern molecular characterisations of degenerative and regenerative processes.

### 4.1 Comparison of ancient and modern doctrines on nervous regeneration

When drawing parallels between the doctrines of Waller, Ranvier, Cajal and the recent theory of nerve regeneration, it is necessary to consider three aspects: 1) particular histological observations, 2) general cellular mechanisms (such as sprouting), 3) the cellular and molecular characterisation of cell interactions at stake in degeneration and regeneration. Consequently, any epistemological analysis comprehending the views of Cajal together with modern conceptions can be beneficial on specific and general issues, in order to show filiations but also, in some cases, to reveal the incommensurability of the views from these close, but distinct, paradigms. In the order of the chronology of degenerative and regenerative processes, such issues may be: 1) the alterations of myelin and more generally all the modifications of Schwann cells, 2) the growth and guidance of axons to the periphery, 3) the myelination of new fibres. However, only the first issue will be addressed in this paper.

### 4.2 Ranvier and Cajal on the early degeneration of Schwann cells and their modifications

From the studies of Ranvier to molecular approaches, through Cajal’s studies and views, we can mention two opposite and intertwined trends. The first is that of collecting extremely precise “details” as Ranvier did, and Cajal and contemporaries even more so. But this whole host of cellular phenomena, apparently independent, were often observed in isolation, often without any glimpse of the causalities between them which gives the wrong impression of fragmented biological mechanisms.

The second trend of molecular biology leads to the discovery of the intracellular signaling pathways engaged in Schwann cells and axons of the central stump. But these studies mainly focus on the early mechanisms of the sprouting of injured axons or on those leading to modified Schwann cells, in a perspective often restricted to one cell type or two, leaving aside the complexity of the environmental milieu, the plasticity of the extracellular matrix, and the diversity of cell types and different functional states of these subtypes, thus forgetting what was praised by the first trend with its own—now out-of-date—techniques. Moreover, this second trend leaves aside not only these aspects considered with a slow dynamic, but also the cell dynamics and the heterogeneity of their behaviours. This is a lack which the first trend obviously highlights but which has also been pointed out only by rare recent studies. For example, one such study demonstrated this type of complexity in modern perspective and with up-to-date techniques, with new specific findings (Rompolas et al., 2012). With this in mind, it appears that the reciprocal evaluation of both trends, with their specific issues, is useful to establish novel forms of concepts relative to nerve degeneration and regeneration and new theoretical aspects.

Evidently, one must start with the critical evaluation of the regeneration concept at the turn of the century by modern views. We find that the fractioning of mechanisms into independent events is clear in Ranvier’s studies when he describes the alterations of myelin, their fragmentation and clearance, quite independently from the multiplication of the Schwann nuclei. In 1913, Cajal also presents these same events quite independently (De Felipe & Jones, 1991, part II, *p*. 83–84). But he gives the hypothesis of Marinesco, and later his own results, concerning the involvement of Schwann cells in an early phagocytic activity eliminating myelin debris (De Felipe & Jones, 1991, part II, *p*. 75), whereas Ranvier only observed phagocytic white blood cells. This idea of phagocytic Schwann cells was in the line of Ranvier’s idea of hypertrophied cells of Schwann (nuclei and sheath observed also separately) but with the new idea of an increased assimilating faculty (De Felipe & Jones, 1991, part II, *p*. 80).

**FIGURE 4:**
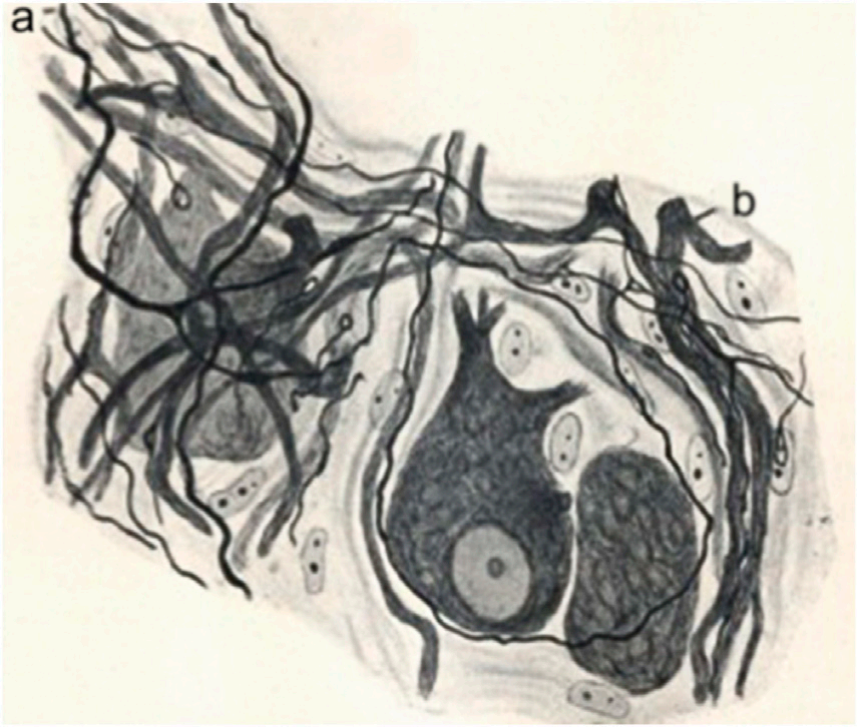
Reinervation of a sympathetic ganglion after a vagus-sympathetic crossed anastomosis. Regenerated preganglionic fibers invade the ganglion. Adapted from de Castro, 1937; reproduced in de Castro, 2016.

**FIGURE 5:**
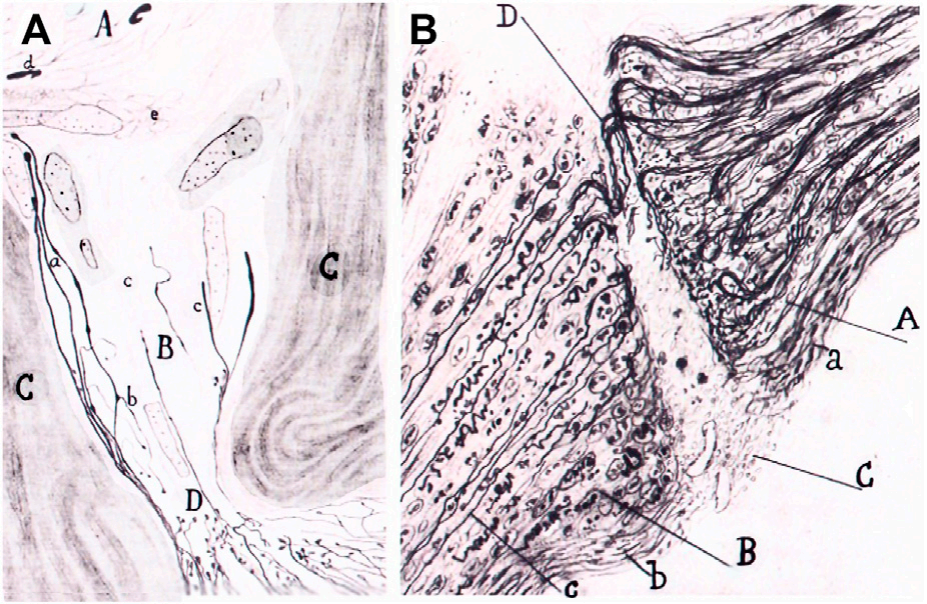
Graft of a piece of the sciatic nerve of a rabbit in the retinal area of the optic nerve. Portion of the sciatic nerve **(A)**; degenerated part of the optic nerve **(B)**; connective tissue of the optic nerve **(C)**; strangulation of the optic nerve produced by the graft **(D)**; new fibers **(a)**; collaterals going backwards **(b)**; connective tissue invading the degenerated optic nerve. **(B)**. Same experiment. Sproutings in the optic nerve **(A)**; portion of the sciatic nerve **(B)**; scar **(C)**; nerve sprouts crossing the scar **(D)**; connective tissue of the optic nerve **(a)**: new neurilemma covering the graft **(b)**; new fiber reaching the graft **(c)**. Adapted from Tello, 1911; reproduced in Martínez-Tello, 2020.

Quite interestingly, Cajal was in this perspective on the way to uniting other events concerning Schwann cells when he interpreted alterations of the Schwann cells as a rejuvenescence, defined as the return to a previous stage (De Felipe & Jones, 1991, part II, *p*. 80), which Cajal correlated with phagocytic activity and the formation of long chains or “protoplasmic bands”. Therefore, what Cajal is building, between the lines of his descriptions, is the beginning of a unified vision of the modifications of Schwann cells, which required the building of the Schwann cell concept at the turn of 20th century (Barbara & Boullerne, 2020). But the idea of rejuvenescence of Cajal was in accord with the ancient idea that inflammation produces cells to return to a sort of embryonic state^36^ which Waller, among others, had also defended. For Cajal, the Schwann cell is first injured, then it undergoes rejuvenescence, cell proliferation and a final differentiation, during the formation of the bands of Büngner and the appearance of myelin in Schwann cells. Such cellular perspectives of Cajal were later developed further by his followers, Fernando de Castro on autonomic ganglia after the work of John Newport Langley (de Castro, 2016: Ros-Bernal & de Castro, 2019), Giuseppe Levi (Figure 4) (Grignolio & de Sio, 2009) and Jorge Francisco Tello Muñoz (Martínez-Tello, 2020), before the work of Rita Levi-Montalacini and others (Figure 5).

Nowadays, molecular analyses have added new dimensions to the unification of Schwann cell alterations, demonstrating for example that they represent a unique reprogramming process. In 2012, an important article addressed this issue in this way: “To what extent are natural transitions in the state of differentiated [Schwann] cells […] governed by specific transcription factors ?” (Arthur-Farraj et al., 2012). This question is in fact asking whether the phenotypic changes of Schwann cells were due or not to a single biological reprogramming mechanism. In the preceding years, French developmental biologist, Nicole Le Douarain and her group, were able to reprogram Schwann cells experimentally into myofibroblasts or glial-melanocytic precursors (Dupin et al., 2003; Real et al., 2005). And the conclusion of the paper by Arthur-Farraj et al. further showed that Schwann cells of the distal stump of a cut nerve *in vivo* expressed the transcription factor c-jun which was necessary to induce an array of phenotypic changes. These included the expression of trophic factors and adhesion molecules, the phagocytosis of myelin and degenerated axons, the formation of the bands of Büngner, with the consequence of reprogramming myelinating and non-myelinating Schwann cells by transdifferentiation^37^ into the states of repair cells and regenerative cells (in the Büngner bands and myelinating cells). This type of discovery clearly established dedifferentiation into states close to glial cell precursors [Arthur-Farraj et al., 2012, (*p*. 643)], redifferentiation and transdifferentiation programs, implying distinctive sub-classes of Schwann cells, with new functional implications in the modern conception of nerve degeneration and regeneration.

### 4.3 Perspectives for the current nervous regeneration model

The example of the changes of Schwann cells during degeneration and regeneration demonstrates how the molecular studies of the signaling pathways substantiate ancient views, such as that of “rejuvenescence” which was already considered an active phenomenon. Other examples concerning the degeneration and the regenerative mechanisms of axons or the changes of extracellular matrix and path finding mechanisms may be analysed in the same way with the same epistemological conclusions.

Nevertheless, additional work is needed to reconcile the studies of the turn of the 20th century with modern issues on regeneration in order to explain the diversity of cellular behaviours in the light of basic and general molecular mechanisms which also have a complexity and diversity of their own with redundancy and vicariance^38^. For example, what molecular events occur in an axon transformed in a large club because it did not reach its target?

Finally, an issue is now raised regarding the common cellular and molecular mechanisms of the reparative regeneration in different tissues (Iismaa et al., 2018). The studies on peripheral nerves presented in the present paper may shed some light on a common regeneration concept. In the same way, a modern common conception may raise new issues on particular reparative regenerations. Such a common concept can be seen as a tool to provide an open perspective bridging together several biological mechanisms involved in regeneration, particularly the occurrence of a short-lived inflammatory reaction inducing cell differentiation reprogramming, transdifferentiation, capillary permeabilization, invasion by blood cells, the formation of a plastic and heterogeneous extracellular matrix, cell proliferations, the involvement of stem cells and progenitors, phenomena of polyploidy, cell migrations up-regulated by the matrix, with retrocontrols of secreted matrix products by migrating cells and cell differentiations and repair.

But there is no doubt that what attracts most of the attention of investigators now concerns the understanding, in the framework of a common modern concept of regeneration, of the blockade phases, as in the central nervous system (Otero, 2018, especially addressing Cajal’s disbelief in the regeneration in the central nervous system), in order to find ways to counter them and induce regeneration in the brain or perfect regeneration as in the case of myocardium, with the ultimate goal of prolonging life.

## Data Availability

The raw data supporting the conclusions of this article will be made available by the authors, without undue reservation.

## Footnotes

1 “ [une] substance dure, […] qui se fait de ce qui abonde de l’aliment de l’os rompu, laquelle le tient et l’aglutine, et avec le temps s’endurcit si fort, que l’endroit de telle glutination se trouve plus ferme et plus dur que l’autre partie non rompue. Car comme la colle sert au bois pour le joindre, semblablement le callus sert aux os rompus pour les joindre et agglutiner ensemble”. All translations by the author, (Malgaigne, 1840, book 13th, chapter 3).

2 “ [une] chair que la nature aura produite dessus : laquelle étant nouvellement engendrée est molle comme fromage nouvellement coagulé, […] [et] avec le temps elle s’endurcit, et se forme en manière de petits grains de grenade, en laquelle on voit la sanie rougeâtre, polie, égalé, glutineuse, non fétide, et puis blanche”, (Malgaigne, 1840, book16th, chapter 34).

3 “ […] d’icelle médulle, et de la propre substance de l’os se fait une résudation crasse et terrestre, dont s’engendre et fait le callus…” (Malgaigne, 1840, book 13th, chapter 29).

4 “ […] tissus compacte et celluleux, ainsi qu’à toutes les parties de la surface divisée en général” (Charmeil, 1821, p. 368).

5 “ […] ce développement de bourgeons qui se fait sur toutes les parties divisées, qui n'est, proprement dit, que l'extension du parenchyme nutritif, se mettant en rapport avec la gélatine pour passer successivement à l'état cartilagineux, puis osseux, développement fait lui-même du système vasculaire, principe formateur de toute création organique” (Charmeil, 1821, p. 369).

6 “[…] quand cette nouvelle substance vient à se raffermir, elle semble changer de nature, elle devient blanche, uniforme, plus ou moins solide, selon les parties qu’elle répare, & elle paroît en quelque sorte informe, si nous la comparons avec la substance des parties qui l’ont fournie”, (Quesnay, 1764, p. 261).

7 See for example the polemics in *Le Mémorial diplomatique* (January 4th 1873, p. 827-828) concerning the publication of the book by J. N. Demarquay, (1874). *De la régénération des organes et des tissus en physiologie et en chirurgie*. Paris: Baillière, 1874. The review of the book mentions the striking divergence of ideas lasting for centuries on animal regeneration (p. 827).

8 Recently, the hypothesis emerged that the history of nerve repair perhaps started during Antiquity. A recently discovered Ottoman surgical manuscript of the 16th century quotes an unknown text of Hippocrates describing the ligature of an injured nerve in a man with a hair (Belen et al., 2009).

9
Ochs (1977) quotes an experimental study by Clarke and Bearn revealing such bands on axons using an old 18th century microscope of the type used by Fontana to observe nerve fibers in the distal stump of an injured nerve (Clarke and Bearn, 1972).

10 Fontana had made the observation of the scar of a reunited injured nerve filled with “cellular tissue” (Fontana, 1784, p. 180: description of nerve fibers, p. 201–203: note on nerve degeneration).

11 Müller’s concern was also due to the use of nitric acid, which he felt unreliable to dissolve surrounding tissues in order to better observe nerve fibers (Müller, 1838, p. 417).

12 Other investigators accepted as true some observations of fibrils before 1830. See also Clarke and Bearn (1972).

13 “Les auteurs qui ont traité de la régénération des nerfs ont subordonné leur manière de voir sur ce sujet à l'opinion qu’ils s’étaient faite de la dégénération, ce qui montre bien, comme je viens de le dire, que c’est la théorie qui les conduisait, même dans l'observation des phénomènes” (Ranvier, 1878b, p. 42-43).

14 “[…] All the debates concerning the reproduction and the regeneration of nerves only focussed on the reproduction of tubes in the scar. All investigators were influenced by what occurs in other tissues, since they only examined the tubes in the scar, with no examination of the peripheral ends. However, the answer to all questions dealing with the reproduction of the nervous substance lies in that part”. Tous les débats qui ont eu lieu par rapport à la reproduction et à la régénération des nerfs, sont seulement sur la reproduction des tuyaux dans la cicatrice. Tous les observateurs, influencés probablement par ce qui se passe dans les autres tissus, se sont bornés à les examiner dans ce lieu, laissant de côté l’examen des bouts périphériques. C’est cependant dans cette partie qu’est la difficulté, c’est là qu’il faut chercher la solution de toutes les questions de reproduction de la substance nerveuse», (Waller, 1852a, p. 4)

15 Waller wasn’t entirely fair either when he had an argument with his French master in microscopy, Alfred Donné, whom he accused of neglecting his role when Donné reported his observations of blood circulation in the tongue of the frog prepared in the way of Waller (Donné, A. (1844). Cours de microscopie. Paris: Baillière, p. 108). See also Sykes (2004), p. 27-29.

16 When visiting London in 1887, Arnemann was opposed to the publication of the study of Cruikshank and he later told Haighton that his anatomical preparation did not show any new nervous substance (Holmes, 1951, p. 46, 50, 52; Arnemann, 1786; Arnemann, 1787).

17 A. Waller chose the model of the tongue of a living frog which was stretched with needles on the microscope stage to study microscopic events. Waller thought the model would allow him to study muscle contraction and nerve degeneration (Waller, 1849a).

18 Waller (185), p. 426) writes: “as we ascend towards the brain the disorganization appears to decrease”.

19 This letter is the memoir published by Waller in French in Bonn (Germany) (Waller, 1852a). Waller writes: “the results of my experiments showed that the old fibres of a divided nerve never recover their initial function and that the reproduction (regeneration) of the nerve does not only occur in the scar, but reaches the terminals”.

20 Waller (1852b), p. 393-394 writes that the new fibres appear as old tubes deprived of their double contours (myelin). But he can get a more accurate description with the use of acetic acid dissolving surrounding tissues showing the nervous mass is rather composed of fibers identical to embryonic ones, pale, with a fine granulated structure and an external membrane with no double contours.

21 Waller (1852b), p. 393 writes: “For the functions [of the cut nerve] to be restored in the distal stump, it is necessary that all old nerve fibers are removed and that completely new ones coming from the central stump emerge in that part as well as all the way to the periphery”.

22 According to this law, nerve fibres elongate from the nerve cells of the nerve centres to the periphery (Kölliker, 1852). For the law of unilateral growth see p. 22 (Section 2.1. *Theorie der Zellenbildung*) and for general ideas on the nervous tissue, see p. 68-70 (Section 3. *Nervengewebe*).

23 Original quotation by Waller: “Il est donc démontré qu’un nerf […] étant coupé, ses nouvelles fibres […] se développent du centre à la circonférence, et non de la circonférence au centre. Je crois qu’il serait superflu d’examiner la question [de savoir] si les fibres de l’adulte ou du jeune animal se développent de la même manière que sur l’embryon, car il est impossible d’admettre que la nature procède autrement dans un cas que [sic] (comme) dans l’autre” (1852c, p. 676-677).

24 See the clinic observations of Sir James Paget (1814-1899) from the 1850s on (Paget, 1863, p. 282; Ochs, 1977, p. 270). Paget uses the expressions of “immediate union” and the “primary adhesion” of nerves.

25 In his lessons, Ranvier extends this list to additional authors quoted in this order: Bruch, Lent, Hjelt, Eulenburg et Landois, Schiff, Philippeaux & Vulpian, Neumann, Erb, Hertz, Laveran, Cossy & Dejérine, Engelmann (Ranvier, 1878a, p. 273). For additional information on Jean-Marie Philippeaux, see Bange and Bange (2010).

26 The Naturmuseum Senckenberg in Frankfurt (Feinsode, 2011).

27 Schiff writes originally: “[…] dans quelques cas, où le galvanisme a révélé à M. Waller le retour des fonctions [du nerf coupé], il a vu, en effet, de véritables fibres régénérées; mais il n’y a pas porté son attention comme il était trop préoccupé de ses prétendues fibres embryonnaires” (Schiff, 1854, p. 451).

28 Schiff quotes the observations by Sir James Paget (Schiff, 1854, p. 449).

29 It took Ramón y Cajal several years before he admitted Ranvier’s ideas on the Schwann cell and its role in the production of myelin (Barbara & Boullerne, 2020; De Felipe & Jones, 1991, part II, p. 44).

30 These morphological elements were not yet considered to be large cellular entities since they occurred as nuclei only covered by a thin layer of protoplasm according to Ranvier.

31 An internode segment occured bewteen two nodes, two concepts developed by Ranvier (Barbara, 2005).

32 Ranvier’s “*cellules lymphatiques*”.

33 For example, Magendie’s observations of red blood cells which he recommended not to do in water.

34 The cell physiology of Brücke, Schultze and Kölliker as studied by François Duchesneau. See for example, Duchesneau (2019).

35 Cajal wrote: “It is only to the talent of such men as Waller and Ranvier that has been able to supply the methodological deficiencies which have led astray many modern histologists of no mean capacity” (De Felipe & Jones, 1991, p. 16).

36 According to J.N. Demarquay, “a law prevails in all the pathological physiology of inflammation […] This law states that inflammation results in the return of adult cells to an embryonic state ; without this transformation, (cell) proliferation cannot occur” ; “une loi domine toute la physiologie pathologique de l'inflammation[…] Cette loi est la suivante: L’inflammation fait repasser la cellule adulte à l'état embryonnaire ; sans cette modification, aucune prolifération ne peut se produire» (Demarquay, 1874), p. 74.

37 Transdifferentiation is a dedifferentiation and redifferentiation of a cell to a differentiated state which differs from the initial one.

38 When a signaling pathway is induced in different cellular subtypes with sometimes different outcomes.
